# Non-COVID Admissions to the ICU After COVID Vaccination: A Multicenter Study

**DOI:** 10.7759/cureus.71534

**Published:** 2024-10-15

**Authors:** Amarja A Havaldar, Kaladhar Sheshala, Raman Kumar, Abhilash Chennabasappa, Ria R Thomas, Sumithra Selvam

**Affiliations:** 1 Critical Care Medicine, St. John's Medical College Hospital, Bengaluru, IND; 2 Critical Care Medicine, Yashoda Hospital, Hyderabad, IND; 3 Anesthesiology, Rajendra Institute of Medical Sciences, Ranchi, IND; 4 Critical Care Medicine, Jagadguru Sri Shivarathreeshwara Medical College and Hospital, Mysuru, IND; 5 Biostatistics, St John's Research Institute, Bengaluru, IND

**Keywords:** bbv152, chadox1 ncov-19, covaxin, covid-19, covishield, guillian barre syndrome, vaccination, vitt

## Abstract

Introduction: The vaccination drive for COVID-19 was launched in India after the authorization of ChAdOx1 nCov-19 (Covishield), an adenoviral vector vaccine, and BBV152 COVID-19 (Covaxin), an inactivated virus vaccine. As stated by the CDC, vaccine-related adverse events can happen. In this study, we aimed to assess the timing of the COVID vaccination and admission diagnosis and their effect on ICU mortality. The time from vaccination can help identify adverse events directly related to vaccination.

Methods: A retrospective cohort study was conducted across four centers in India. Patients who took the first or second dose of any vaccines and were admitted with non-COVID illness to the ICU were included in the study. Patients were categorized based on the time interval from vaccination as ≤42 days or >42 days. The primary outcome was ICU mortality. The secondary outcomes were the length of ICU stay and duration of mechanical ventilation.

Results: A total of 175 patients were included in the study. The mean age was 53.49 (15.89) years, and 61.14% were males. The ICU mortality was 24.57% (18.38% to 31.63%). Thromboembolic events such as acute coronary syndrome (ACS), cerebrovascular accident (CVA), and mesenteric ischemia were seen in 7.43%, 7.43%, and 1.14% of patients, respectively. Six patients (3.43%) developed neuromuscular illness. The mortality was higher in patients >66 years, followed by ≤35 years of age when admitted ≤42 days of vaccination (p=0.008). The mortality was higher in cerebrovascular disorders and was clinically significant (p<0.001).

Conclusion: Patients developed thromboembolic events and neuromuscular diseases requiring ICU admission post-COVID vaccination. We observed a significantly higher mortality in the age groups >66 years and ≤35 years when admitted within 42 days of COVID vaccination. Patients admitted with cerebrovascular diseases also had higher mortality.

## Introduction

Various strategies were used to contain the pandemic and to treat COVID-19 patients. With the emergency authorization of vaccines after initial phase III trials, the vaccination program was rolled out on 16th January 2021, in India [1]. Initially, vulnerable groups and frontline workers were given the vaccination, and over the next few months, it was made available to the general public.

There were concerns about the effectiveness and adverse events related to vaccination. The initial study by Kewan et al. reported adverse events after COVID vaccination requiring emergency department visits within 10 days [2]. The case series, including five patients, described the development of vaccine-induced thrombocytopenia (VITT) after ChAdOx1 nCov-19 (Covishield) [3]. As per the CDC report, the complications can occur within six weeks, i.e., 42 days, following vaccination [4]. There is limited information from India about adverse events after vaccination, and it is only restricted to case reports, system-specific events, or limited to self-reporting of adverse events by the people [5-6]. Information about admissions after COVID vaccination is still important to identify any short-term or long-term effects directly related to vaccination for safety and quality purposes. We aimed to see the association between the time interval between COVID vaccination and admission diagnosis and their effect on ICU mortality. 

## Materials and methods

Design and settings

This study was conducted from 1st April 2021 to 31st December 2021. As this was a retrospective cohort study, a waiver for informed consent was obtained from the Institutional Ethics Committee (IEC) (approval no. 149/2021) of St. John’s Medical College Hospital. The study was registered with the Clinical Trial Registry India (CTRI; 2021/07/034587) on 05/07/2021. The IEC approvals from the respective participating centers were obtained. A total of four centers were included from India.

The Strengthening the Reporting of Observational Studies in Epidemiology (STROBE) guidelines were followed. After IEC approval from each center, the data collection process was initiated. This study is a part of the Postcovac study [7].

Participants

The patients who received the first or second dose of any of the COVID vaccines were included in the study. The baseline characteristics of the patients were collected. Information about the type of vaccine and the number of doses received was collected. Time from vaccination was calculated as the difference between the timing of the vaccination, i.e., either the first or second dose, whichever was the latest, and the ICU admission for non-COVID illness. The time from vaccination was divided into two categories: ≤42 days and >42 days. Acute physiology and chronic health evaluation (APACHE II) and sequential organ failure assessment (SOFA) scores were calculated. The admission diagnosis was classified into 10 subgroups. The primary outcome was ICU mortality. The secondary outcomes were the length of ICU stay and duration of mechanical ventilation.

Data analysis

Statistical analysis was done using STATA version 15 (StataCorp LLC, College Station, TX, USA). Continuous variables were presented as mean (standard deviation (SD)) and median (interquartile range (IQR)) as applicable. Categorical variables were presented as percentages. Continuous variables were analyzed by an independent t-test or Mann-Whitney U test as applicable. The chi-square test was used to test the association between categorical variables. Logistic regression was done to test the association between ICU mortality and time from vaccination adjusted for age, gender, presence of comorbidities, and APACHE II score in different models. The p-value of <0.05 was considered statistically significant.

## Results

Among the 506 patients who were screened, 175 vaccinated patients were enrolled in the study based on the inclusion criteria from four centers. The clinical characteristics of the study population are presented in Table 1. The mean age was 53.49 ± 15.89 years, and 61.14% were males. The median APACHE II and SOFA scores were 17 (11 to 24) and 7 (5 to 9), respectively (Table 1). Information about the type of vaccine received was collected. The median time from vaccination to ICU admission was 55 (25 to 85) days, and 41.7% were admitted within ≤42 days of vaccination.

**Table 1:** Clinical characteristics of the study population Values are presented as n (%), ¥ mean (SD), and median (25th and 75th percentiles). CKD: Chronic kidney disease, CVA: Cerebrovascular accident, IHD: Ischemic heart disease, COPD: Chronic obstructive pulmonary disease, TB: Tuberculosis, APACHE: Acute physiology and chronic health evaluation, SOFA: Sequential organ failure assessment

Parameters	Values (total n=175)
Age^¥^	53.49 ± 15.89
≤35	27 (15.43)
36 to 50	43 (24.57)
51 to 60	60 (34.29)
≥66	45 (25.71)
Gender	
Male/female	107/68 (61.14/38.86)
Time from vaccination to hospital admission	55 (25, 85)
≤42 days	73 (41.7%)
>42 days	102 (58.3%)
Vaccination	
Single dose /Two doses	92/83 (52.57/47.43)
Type of vaccine	
Covishield vs. Covaxin	142/27 (84.02/15.98)
Comorbidities	
Diabetes mellitus	70 (40)
Hypertension	78 (44.57)
CKD	16 (9.14)
CVA	15 (8.57)
IHD	25 (14.29)
COPD	8 (4.57)
Bronchial asthma	3 (1.71)
TB	4 (2.29)
Immunosuppressants	8 (4.57)
Malignancy	3 (1.71)
Scores	
APACHE II score	17 (11-24)
SOFA score	7 (5-9)

The overall time taken for admission diagnosis of the patients in the ICU and the time from vaccination for ≤42 days and >42 days are presented in Table 2. The most common admission diagnosis was sepsis (38.86%). Acute coronary syndrome (ACS) (7.43%), cerebrovascular accident (CVA) (7.43%), and mesenteric ischemia (1.14%) were the possible thromboembolic events (16%). Two patients had mesenteric ischemia after 64 and 100 days after vaccination, respectively. Six patients (3.43%) had neuromuscular illness (Guillian Barre syndrome (GBS), myasthenia gravis, or any other), of which three patients presented with GBS. Among these three patients, GBS was diagnosed within six weeks in two patients and within two days after the second dose in one patient. One patient developed demyelinating polyradiculopathy with brainstem involvement (atypical GBS) 15 days after the vaccination. Two patients developed myasthenia gravis six weeks after vaccination. Intracranial bleeding was seen in 6.86% of the patients. There was no significant difference in the admission diagnosis, length of ICU stay, or duration of mechanical ventilation between the time from vaccination in ≤42 and >42 days. Also, admission diagnosis was comparable to the type of vaccine received.

**Table 2:** Admission diagnosis and timing from vaccination Values are presented as n (%) and p-value from the chi-square test of association. ACS: Acute coronary syndrome, CVA: Cerebrovascular accident

Admission diagnosis	All (total n=175)	Time from vaccination ≤42 days (total n=73)	Time from vaccination >42 days (total n=102)	p-value
Mortality	24.57%	30.14%	20.59 %	0.148
ACS	13 (7.43)	4 (5.48)	9 (8.82)	0.165
CVA	13 (7.43)	6 (8.22)	7 (6.86)	
Mesenteric ischemia	2 (1.14)	0 (0)	2 (1.96)	
Neuromuscular diseases	6 (3.43)	2 (2.74)	4 (3.92)	
Intracranial bleeding	12 (6.86)	5 (6.85)	7 (6.86)	
Sepsis	68 (38.86)	23 (31.51)	45 (44.12)	
Tropical fever	17 (9.71)	12 (16.44)	5 (4.90)	
Other diagnosis	30 (17.14)	12 (16.44)	18 (17.65)	
Hepatic	9 (5.14)	6 (8.22)	3 (2.94)	
Renal	5 (2.8)	3 (4.11)	2 (1.96)	
Length of ICU stay	7 (3-13)	6 (3-10)	7 (4-15)	0.072
Duration of mechanical ventilation	4 (3-8)	4 (3-5)	4 (3-8)	0.762

The overall mortality was 24.57% (18.38% to 31.63%). The association of clinical characteristics between survivors and non-survivors is presented in Table 3. Time from vaccination to ICU admission was significantly lower in non-survivors compared to survivors (p=0.033). Although statistically not significant, the proportion of mortality (30.14%) was higher in patients admitted within 42 days post-vaccination as compared to those admitted after 42 days of vaccination (20.59%), p=0.148. The APACHE and SOFA scores were significantly higher in non-survivors (p<0.001) (Table 3). Adjusted for age, gender, diagnosis, and the presence of comorbidities (diabetes mellitus and hypertension), an increased number of days after vaccination had a lower risk of mortality (adjusted odds ratio (AOR): 0.989, 95% CI, 0.980-0.998, p=0.020). After adjusting for APACHE II along with the above-mentioned variables in the model, the time from vaccination remained significantly associated with mortality (AOR: 0.990, 95% CI, 0.980-0.999, p=0.042).

**Table 3:** Association of clinical characteristics between survivors and non-survivors ^¥^Mean (SD): The independent sample t-test was used for comparison; *Median (IQR): Mann-Whitney U test was used for comparison. Values are presented as n (%). CKD: Chronic kidney disease, CVA: Cerebrovascular accident, IHD: Ischemic heart disease, COPD: Chronic obstructive pulmonary disease, TB: Tuberculosis

Parameters	Survivors (total n=132)	Non-survivors (total n=43)	p-value
Age^¥^	52.44 (15.29)	56.72 (17.42)	0.126
Gender: Male/female	80/52 (60.61/39.39)	27/16 (62.79/37.21)	0.799
Time from vaccination to hospital admission (days)	58.5 (28, 87)	37 (15, 79.0)	0.033
Time from vaccination ≤42 days	51 (69.86)	22 (30.14)	0.148
Time from vaccination >42 days	81 (79.41)	21 (20.59)	
Any vaccination: Single dose/two Doses	64/68 (48.48/51.52)	28/15 (65.12/34.88)	0.058
Type of vaccine: Covishield vs. Covaxin	108/20 (84.38/15.63)	34/7 (82.93/17.07)	0.826
Comorbidities			
Diabetes mellitus	52 (39.39)	18 (41.86)	0.774
Hypertension	55 (41.67)	23 (53.49)	0.176
CKD	11 (8.33)	5 (11.63)	0.515
CVA	13 (9.85)	2 (4.65)	0.290
IHD	16 (12.12)	9 (20.93)	0.152
COPD	7 (5.30)	1 (2.33)	0.417
Bronchial asthma	3 (2.27)	0 (0)	0.319
TB	3 (2.27)	1 (2.33)	0.984
Immunosuppressants	4 (3.03)	4 (9.30)	0.087
Malignancy	3 (2.27)	0 (0)	0.319
Scores			
APACHE II score*	14 (9-22)	19.5 (17-24)	<0.001
SOFA score*	6 (4-8.5)	9 (7-11)	<0.001

**Figure 1:**
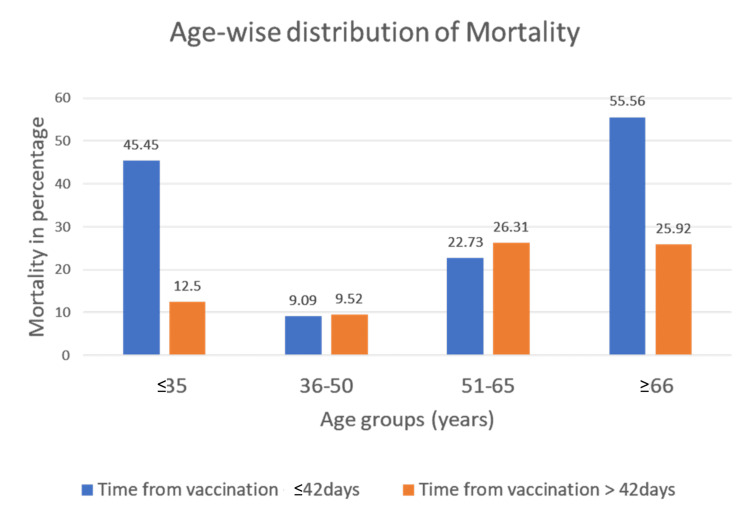
Age-wise distribution of mortality

In the subgroup analysis of patients admitted within 42 days post-vaccination, the proportion of mortality was significantly higher in the ≤35 years and >66 years age groups compared to the middle-aged group of patients (p=0.008). After 42 days of vaccination, there was no significant association noted between age categories and mortality (Figure 1). 

Among non-survivors in the ≤35years of age group and admitted within 42 days of vaccination, the admission diagnoses were intracranial bleeding, liver failure, fever with demyelinating polyradiculopathy (atypical GBS) with brainstem involvement, and tropical fever with thrombocytopenia (platelet factor 4 (PF4) antibodies were negative and the patient had brainstem dysfunction). One patient diagnosed with pre-eclampsia developed puerperal sepsis.

## Discussion

In our study, 175 patients were admitted post-vaccination with non-COVID illness during the study period. The admission diagnosis possibly related to vaccination includes thromboembolic events and neuromuscular illness. Higher mortality was observed in the age group >66 years and ≤35 years when the time from vaccination was ≤42 days. The ICU mortality was 24.57%.

The common complications reported in different studies are vaccine-induced immune thrombotic thrombocytopenia (VITT), thromboembolic complications, and neurological complications. Vaccine-induced immune thrombotic thrombocytopenia was reported within one week of receiving the vector-based Covishield vaccine, and PF4 antibodies were positive [8-12]. A single-center study evaluated coronary thrombosis post-vaccination within three months, but the causality could not be determined [6]. Systematic review and case series evaluating thromboembolic complications post-vaccination showed the median duration for the development of these complications was 10.8+7.8 days and PF4 antibodies were present in 78.6% of patients [4,13,14].

There are different neurological complications described after vaccination [15]. Guillian Barre syndrome is a known complication seen after various vaccinations, such as H1N1 [16]. This complication can occur up to six to eight weeks post-vaccination. Similar case reports are seen post-COVID vaccination [17,18]. We observed neuromuscular illness in six patients (3.16%).

The median time from vaccination to hospitalization was 55 (22 to 85) days, and for ACS, CVA, neuromuscular illness, intracranial bleeding, and mesenteric ischemia, it was 75 (38 to 99), 44 (28 to 56), 50 (36 to 54), 94.5 (17 to 138.50), and 82 (64 to 100) days, respectively. Based on the published literature, the reported thromboembolic events are observed within seven to 10 days, and neuromuscular illness in six to eight weeks [4,16]. In our study, neuromuscular illness is possibly related to vaccination; however, causality could not be determined. Time from vaccination alone was the significant factor in logistic regression after adjusting for covariates, including APACHE II.

In our study, age-wise mortality observed in ≤35-year-old patients was 45.45% when the time from vaccination was ≤42 days. The reasons for the higher mortality need to be studied in the larger population. There are reports describing a higher incidence of myocarditis and mortality in a younger age group after mRNA vaccination [19].

We classified admission diagnosis based on the International Classification of Diseases (ICD) 10 and compared the mortality with available literature [20]. The most common ICD10 code was I60-69, which includes cerebrovascular diseases. As compared to other illnesses, the mortality in this group was higher as compared to the published data (31.82% vs. 11.41%, p=0.001). This needs further evaluation in a larger cohort.

There are various strengths to our study. It is one of the studies on the effect of timing from COVID vaccination and admission diagnosis in patients admitted to the ICU with non-COVID illness. The available published information regarding the adverse events is limited [17,21]. Our study gives an overview of admission diagnosis in vaccinated patients, focusing more on thromboembolic and neuromuscular diseases.

There were certain limitations to our study. This is a retrospective study. We included patients vaccinated with Covishield and Covaxin; hence, the side effects or adverse events observed in this study will not apply to patients who received different types of vaccines. Patients who didn’t report to the hospital due to minor side effects or had fatal outcomes were not included in this study. Hence, this may not give information about the actual incidence of adverse events. The comparison between the unvaccinated cohort admitted during the same time period would have helped in comparing the incidence of adverse events. We compared a cohort of patients admitted before COVID to overcome this limitation [20]. As this vaccine is new, follow-up of the vaccinated cohort is necessary to know the long-term effects of the vaccination, if any.

## Conclusions

This study shows that adverse events, although rare, can occur after vaccination. We observed higher mortality in extremes of age group when admitted within 42 days of vaccination. We also observed higher mortality in patients with cerebrovascular diseases. A large population-based study is required to confirm these findings.

## References

[1] Kumar VM, Pandi-Perumal SR, Trakht I, Thyagarajan SP (2021). Strategy for COVID-19 vaccination in India: the country with the second highest population and number of cases. NPJ Vaccines.

[2] Kewan T, Flores M, Mushtaq K (2021). Characteristics and outcomes of adverse events after COVID-19 vaccination. J Am Coll Emerg Physicians Open.

[3] Coronavirus Disease 2019 (COVID-19) | COVID-19 | CDC. https://www.cdc.gov/covid/?CDC_AAref_Val=https://www.cdc.gov/coronavirus/2019-ncov/vaccines/expect/.

[4] Schultz NH, Sørvoll IH, Michelsen AE (2021). Thrombosis and thrombocytopenia after ChAdOx1 nCoV-19 vaccination. N Engl J Med.

[5] Chakraborty A, Reval N, Kamath L (2022). Adverse events following COVID-19 vaccination in selected apartments in Bangalore, India. Cureus.

[6] Showkathali R, Yalamanchi R, Narra L (2022). Coronary thrombo-embolic events after Covid-19 vaccination — a single centre study. Indian Heart J.

[7] Havaldar AA, Prakash J, Kumar S (2022). Demographics and clinical characteristics of COVID-19-vaccinated patients admitted to ICU: a multicenter cohort study from India (Postcovac study-COVID group). Indian J Crit Care Med.

[8] Greinacher A, Thiele T, Warkentin TE, Weisser K, Kyrle PA, Eichinger S (2021). Thrombotic thrombocytopenia after ChAdOx1 nCov-19 vaccination. N Engl J Med.

[9] Sharifian-Dorche M, Bahmanyar M, Sharifian-Dorche A, Mohammadi P, Nomovi M, Mowla A (2021). Vaccine-induced immune thrombotic thrombocytopenia and cerebral venous sinus thrombosis post COVID-19 vaccination: a systematic review. J Neurol Sci.

[10] Wiedmann M, Skattør T, Stray-Pedersen A (2021). Vaccine-induced immune thrombotic thrombocytopenia causing a severe form of cerebral venous thrombosis with high fatality rate: a case series. Front Neurol.

[11] Bilotta C, Perrone G, Adelfio V, Spatola GF, Uzzo ML, Argo A, Zerbo S (2021). COVID-19 vaccine-related thrombosis: a systematic review and exploratory analysis. Front Immunol.

[12] Wills A, Swallow G, Kirkman MA, Rajan K, Subramanian G (2022). Arterial and venous thrombotic stroke after ChAdOx1 nCoV-19 vaccine. Clin Med (Lond).

[13] Mani A, Ojha V (2022). Thromboembolism after COVID-19 vaccination: a systematic review of such events in 286 patients. Ann Vasc Surg.

[14] Thaler J, Ay C, Gleixner KV (2021). Successful treatment of vaccine-induced prothrombotic immune thrombocytopenia (VIPIT). J Thromb Haemost.

[15] Corrêa DG, Cañete LA, Dos Santos GA, de Oliveira RV, Brandão CO, da Cruz LC Jr (2021). Neurological symptoms and neuroimaging alterations related with COVID-19 vaccine: cause or coincidence?. Clin Imaging.

[16] De Wals P, Deceuninck G, Toth E (2012). Risk of Guillain-Barré syndrome following H1N1 influenza vaccination in Quebec. JAMA.

[17] McKean N, Chircop C (2021). Guillain-Barré syndrome after COVID-19 vaccination. BMJ Case Rep.

[18] Introna A, Caputo F, Santoro C, Guerra T, Ucci M, Mezzapesa DM, Trojano M (2021). Guillain-Barré syndrome after AstraZeneca COVID-19-vaccination: a causal or casual association?. Clin Neurol Neurosurg.

[19] Nafilyan V, Bermingham CR, Ward IL (2023). Risk of death following COVID-19 vaccination or positive SARS-CoV-2 test in young people in England. Nat Commun.

[20] Varma MMK, Krishna B, Sampath S (2019). Secular trends in an Indian intensive care unit-database derived epidemiology: the stride study. Indian J Crit Care Med.

[21] Wong HL, Hu M, Zhou CK (2022). Risk of myocarditis and pericarditis after the COVID-19 mRNA vaccination in the USA: a cohort study in claims databases. Lancet.

